# Efficacy of chemotherapy after progression during or following PARPi exposure in ovarian cancer☆

**DOI:** 10.1016/j.esmoop.2024.103694

**Published:** 2024-09-03

**Authors:** A. Xu-Vuillard, C. Guerin-Charbonnel, F. Bocquet, S. Cheeseman, P.M. Kubelac, M. Zenatri, G. Hall, P. Achimas-Cadariu, B. Hanvic, H. Fenton, A.-M.-L. Sturz-Lazăr, P. Augereau, I.R. Coquard, A. Leary, J.-S. Frenel

**Affiliations:** 1 Medical Oncology Department, Gustave Roussy, Villejuif; 2 Sorbonne Université, Paris; 3 Department of Biostatistics and Analytics, Institut de Cancérologie de L’Ouest, Nantes; 4 Data Factory, Institut de Cancérologie de L’ouest, Nantes, France; 5 Leeds Cancer Center, Leeds Teaching Hospitals NHS Trust, Leeds, UK; 6 The Oncology Institute Prof. Dr Ion Chiricuta, Kluj-Napoca, Romania; 7 Medical Oncology Department, Institut de Cancérologie de L’Ouest, Saint-Herblain; 8 Medical Oncology Department, Centre Leon Berard, Lyon, France; 9 Oncology Evidence Network, IQVIA, London, UK; 10 City Hospital, Timisoara, Romania; 11 Medical Oncology Department, Institut de Cancérologie de L’Ouest, Angers, France; 12 University of Medicine and Pharmacy Iuliu Hatieganu, Kluj-Napoca, Romania

**Keywords:** advanced ovarian cancer, PARPi, platinum-based chemotherapy, BRCA

## Abstract

**Background:**

Poly(ADP-ribose) polymerase inhibitors (PARPis) improved advanced ovarian cancer treatment. Most patients progress during or following PARPi exposure, however, with concerns about sensitivity of subsequent chemotherapy.

**Patients and methods:**

In this international cohort study, we evaluated the efficacy of a subsequent chemotherapy following PARPi exposure in high-grade ovarian carcinoma patients. Endpoints included progression-free survival (PFS), overall survival and a multivariable Cox model was built to identify factors influencing PFS.

**Results:**

We included 291 patients from four international centers treated between January 2002 and December 2021. The median number of previous chemotherapy was 1 (1.0-7.0), the median duration of PARPi exposure was 6.5 months (0.2-54.3 months). PARPi was used in first line in 14.1% patients. Most progressions occurred under PARPi exposure (89.1%). A *BRCA* pathogenic variant was identified in 130 patients (44.7%), absent in 157 patients (54.0%), and undocumented in 4 patients (1.4%). Platinum-based CT (PBC) and non-PBC were administered as subsequent treatments in, respectively, 182 patients (62.5%) and 109 patients (37.5%). Multivariable analyses showed that platinum-free interval (PFI) >6 months [adjusted hazards ratio (HR), 0.52; 95% confidence interval (CI) 0.39-0.70] and type of initial surgery (adjusted HR, 1.41; 95% CI 1.07-1.87; interval or closing surgery versus primary surgery) were associated with PFS, independent of *BRCA* status or line of therapy (≥2 versus 1). In patients with a PFI >6 months, PBC was numerically associated with the best PFS (adjusted HR, 0.68; 95% CI 0.46-1.01).

**Conclusion:**

This is the largest real-world study assessing the efficacy of subsequent chemotherapy in patients progressing during PARPi exposure. The patients have poor outcomes. PBC is the best option in patients progressing on PARPi and eligible for PBC rechallenge (PFI >6 months).

## Highlights

• Patients progressing under poly(ADP-ribose) polymerase inhibitors (PARPis) have a poor outcome.
• Platinum-based chemotherapy (PBC) seems the best option after PARPi and in patients eligible for PBC rechallenge.
• PFI >6 months remains a significant prognostic factor in patients progressing under PARPi.

## Introduction

Ovarian cancer is the most fatal gynecological cancer in Europe, affecting 66 693 new patients in Europe in 2020 and causing 44 503 deaths.^1^ Its prognosis has been significantly improved with the introduction of poly(ADP-ribose) polymerase inhibitors (PARPis) maintenance after platinum-based chemotherapy (PBC),^2^ considering the synthetic lethality in tumors with homologous recombination deficiency (HRD).^3^^,^^4^ PARPis were initially approved as maintenance in patients with partial or complete response to PBC for platinum-sensitive relapse and continued until intolerance or disease progression.^5^, ^6^, ^7^ PARPis have now been indicated as first-line maintenance with niraparib for 3 years^8^ and olaparib for 2 years in patients with BRCA1/2-mutated ovarian cancer,^9^ or olaparib plus bevacizumab in patients with HRD-positive ovarian cancer (PAOLA-1).^10^ Although progression-free survival (PFS) significantly improved with PARPis, 55%-85% patients will ultimately relapse.^11^^,^^12^ The optimal management for patients progressing following PARPi exposure remains unclear.^13^ Particularly, for patients progressing during PARPi, cross-resistance mechanisms between PARPi and platinum have raised concerns.^14^ A retrospective series has reported less benefit from PBC rechallenge.^15^^,^^16^ Considering the rapid increase in this patient population, survival outcomes, prognostic markers, and best subsequent treatment options should be identified in these patients in real life.

## Material and methods

### Study design

This noninterventional, retrospective, international study was conducted to assess the outcome of ovarian cancer patients treated with chemotherapy after prior PARPi exposure in a maintenance setting. The patients were diagnosed and treated between January 2003 and December 2021, and the data were compiled until the cut-off date (December 2023), death, or date of last contact (if lost to follow-up). All patients treated for high-grade ovarian carcinoma progressing following or during PARPi and who received an immediate subsequent line of chemotherapy were selected in the four participating centers (Institut de Cancérologie de l’Ouest, France; Leeds Teaching Hospitals NHS Trust, UK; Institut Gustave Roussy, France; The Oncology Institute Prof. Dr Ion Chiricuta, Romania). PARPi could be administered in the first-line or later setting. We collected the demographic and clinical data of patients from their electronic health records. No formal dedicated informed consent was required owing to the use of deidentified data. All patients, however, did not oppose to the reuse of their electronically recorded data. An independent ethics committee (Comité D’Ethique du CHU d’Angers, 2022-135) approved this analysis. The Institut de Cancérologie de l’Ouest has committed to the French Commission Nationale de l’Informatique et des Libertés to comply with the Declaration of Helsinki. This project was registered in the public directory maintained by the National Institute for Health Data. This study followed the Strengthening the Reporting of Observational Studies in Epidemiology (STROBE) reporting guidelines.

### Objectives

This study aimed to describe the outcomes of patients treated with chemotherapy following PARPi exposure in the whole population and subgroups, based on *BRCA* status, platinum-free interval (PFI), and PARPi administration line (≥2 versus 1). The primary endpoint was PFS defined as the time between the starting date of the subsequent line of chemotherapy following PARPi exposure and the date of disease progression or death. The chemotherapies received following progression were classified into PBC and non-PBC (nPBC). Progression was defined according to local practice, involving radiological, clinical, or biological progression. Patients who survived and did not progress during analysis were censored at their last follow-up. Overall survival (OS) was the secondary endpoint and defined as the time between the starting date of subsequent chemotherapy after PARPi (baseline) and the date of death from any cause or last contact (censored data).

### Statistical analysis

We described patient characteristics, pathological characteristics, and treatment types (surgery, chemotherapy, and targeted therapy). Qualitative and quantitative variables were presented as number (%), and median (range) and mean ± standard deviation, respectively. Survival outcomes, with a 95% confidence interval (CI), were estimated using the Kaplan–Meier method and compared using the log rank test. Univariable and multivariable Cox analyses were carried out to determine the impact of PFI (≤6 or >6 months), *BRCA* status, line of therapy of PARPi administration (1 or ≥2), and initial surgery timing (primary versus interval/closing versus none) on PFS, based on patients with known values for all of those parameters.

## Results

### Patients, tumor characteristics, and initial treatments

We included 291 patients from the four centers treated from January 2003 to December 2021. Supplementary Figure S1, available at https://doi.org/10.1016/j.esmoop.2024.103694 shows the flowchart. Table 1 summarizes the baseline characteristics of patients. Briefly, the median age at the start of the first chemotherapy following PARPi exposure was within 60-70 years. A *BRCA* pathogenic variant was identified in 130 (44.7%) patients (112 germline/18 somatic), absent in 157 (54.0%) patients, and undocumented in 4 patients (1.4%). The median number of previous lines of chemotherapy was 1 (range, 1-7). PARPi was administered as maintenance treatment of first, second, third, and fourth lines of chemotherapy in 14.1%, 50.9%, 22.3%, and 12.7% of patients, respectively. The median duration of PARPi exposure was 6.5 months (0.2-54.3 months), 6.3 months (0.2-54.3 months), and 10.4 months (2.7-42.7 months) in the whole population, relapse setting, and first-line setting, respectively. Disease progression occurred during PARPi therapy in 89.1% of patients (*n* = 253), whereas progression occurred after a median PARPi-free interval of 6.4 months (3.0-82.9 months) in 10.9% of patients (*n* = 31). The delay between PARPi and subsequent chemotherapy was unknown for seven patients.

**Table 1:** Characteristics of patients

	Whole population (*N* = 291)	Platinum-based chemotherapy following PARPi (*n* = 182)	Non-platinum-based chemotherapy following PARPi (*n* = 109)
**Year of diagnosis**			
Median (min-max)	2015 (2007-2021)	2015 (2003-2021)	2015 (2007-2021)
**Initial performance score, *n* (%)**			
0	87 (36.6)	46 (31.1)	41 (45.6)
1	85 (35.7)	52 (35.1)	33 (36.7)
2	55 (23.1)	43 (29.1)	12 (13.3)
3	11 (4.6)	7 (4.7)	4 (4.4)
Missing	53 (-)	34 (-)	19 (-)
**Initial FIGO, *n* (%)**			
I	4 (1.4)	1 (0.5)	3 (2.8)
II	9 (3.1)	5 (2.7)	4 (3.7)
III	215 (73.9)	139 (76.4)	76 (69.7)
IV	63 (21.6)	37 (20.3)	26 (23.9)
**Histological type, *n* (%)**			
Serous	272 (93.5)	170 (93.4)	102 (93.6)
Endometrioid	5 (1.7)	2 (1.1)	3 (2.8)
Other carcinoma	14 (4.8)	10 (5.5)	4 (3.7)
***BRCA1* and/or *BRCA2* pathogenic variant, *n* (%)**			
Germline	112 (38.5)	85 (46.7)	27 (24.8)
Somatic	18 (6.2)	14 (7.7)	4 (3.7)
None	157 (54.0)	80 (44.0)	77 (70.6)
Unknown	4 (1.4)	3 (1.6)	1 (0.9)
**Line of first exposure to PARPi, *n* (%)**			
1	41 (14.1)	35 (19.2)	6 (5.5)
2	148 (50.9)	89 (48.9)	59 (54.1)
3	65 (22.3)	34 (18.7)	31 (28.4)
≥4	37 (12.7)	24 (13.2)	13 (11.9)
**Surgery timing, *n* (%)**			
Initial	96 (33.4)	62 (34.8)	34 (31.2)
Interval/closing	160 (55.7)	96 (53.9)	64 (58.7)
No surgery	31 (10.8)	20 (11.2)	11 (10.1)
Missing	4 (-)	4 (-)	0 (-)
**Platinum-free interval, *n* (%)**			
≤6 months	73 (26.1)	11 (6.2)	62 (60.2)
6-12 months	87 (31.1)	57 (32.2)	30 (29.1)
>12 months	120 (42.9)	109 (61.6)	11 (10.7)
Missing	11 (-)	5 (-)	6 (-)
**Interval from PARPi exposure, *n* (%)**			
<3 months	253 (89.1)	155 (87.6)	98 (91.6)
>3 months	31 (10.9)	22 (12.4)	9 (8.4)
Missing	7 (-)	5 (-)	2 (-)
**Duration of exposure to PARPi (months)**			
Mean (SD)	10.4 (9.7)	13.8 (10.1)	4.8 (5.6)
Median (min-max)	6.5 (0.2-54.3)	10.6 (0.4-54.3)	3.0 (0.2-39.1)
Missing	7 (-)	5 (-)	2 (-)

FIGO, International Federation of Gynecology and Obstetrics; PARPi, poly(ADP-ribose) polymerase inhibitor; SD, standard deviation.

### Treatments received following PARPi progression

The median interval between the last platinum and the start of chemotherapy (PFI) following PARPi progression was 9.9 months (range: 1.5-90.3 months), with 73 (26%), 87 (31%), and 120 (43%) patients starting the next line of chemotherapy at ≤6, 6 to ≥12, and >12 months, respectively. Subsequently, 182 (62.5%) and 109 (37.5%) patients received PBC and nPBC regimens, respectively. The characteristics of patients are shown in Table 1. The regimen of chemotherapy (PBC or nPBC) was strongly associated with the PFI (*P* < 0.0001 Fisher’s exact test). PBC was administered mostly in combination (135/182) while nPBC was mostly administered as monochemotherapy (98/109 patients), with the most administered being paclitaxel (*n* = 38). Chemotherapy regimen details are shown in Supplementary Table S1, available at https://doi.org/10.1016/j.esmoop.2024.103694.

### Outcomes of patients with subsequent chemotherapy in the whole population

**Figure 1:**
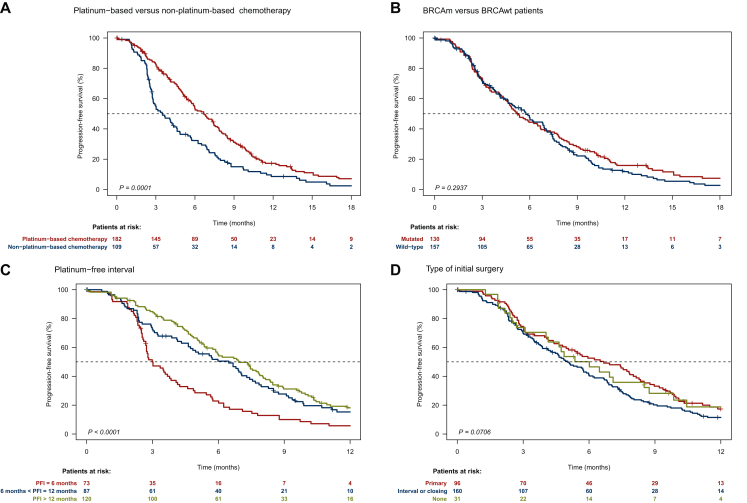
Kaplan–Meier curves for PFS based on chemotherapy received after PARPi (A), *BRCA* status (B), platinum-free interval (C), and type of initial surgery (D). PARPi, Poly(ADP-ribose) polymerase inhibitor; PFI, platinum-free interval; PFS, progression-free survival.

**Figure 2:**
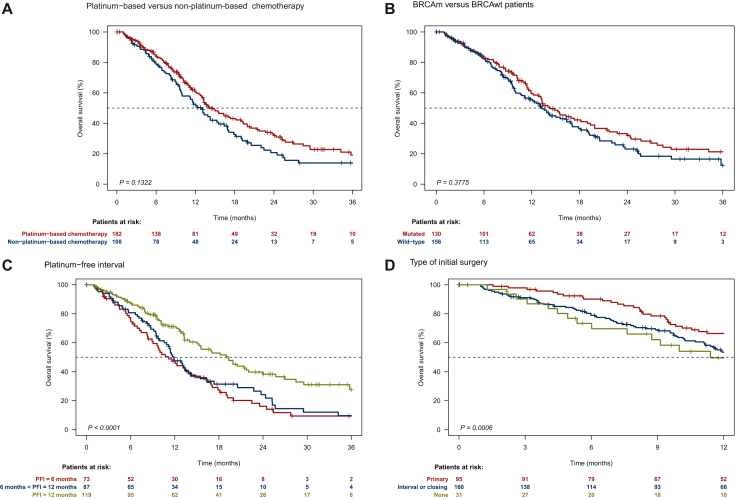
Kaplan–Meier curves for OS based on chemotherapy received after PARPi (A), *BRCA* status (B), platinum-free interval (C), and type of initial surgery (D). OS, overall survival; PARPi, poly(ADP-ribose) polymerase inhibitor; PFI, platinum-free interval.

With a median follow-up of 25.3 months (95% CI 23.0-31.7 months), 258 patients progressed, and 190 patients died. The median PFS and OS from initiation of chemotherapy following PARP exposure in the entire population were 5.6 months (95% CI 4.8-6.5 months) and 13.6 months (95% CI 12.6-16.4 months), respectively. PFI with a 6 month threshold, chemotherapy regimen (PBC or nPBC) was strongly associated with PFS, whereas there was a trend toward the type of initial surgery and no association with the *BRCA* status (Figure 1 and Table 2). PFI and type of initial surgery were associated with OS (Figure 2 and Table 2).

**Table 2:** Survival estimates and comparison for patients in the whole population

	PFS			OS		
	Events/all	Median (95% CI), months	*P* (log rank)	Events/all	Median (95% CI), months	*P* (log rank)
**Type of chemotherapy following PARPi**						
PBC	158/182	6.7 (5.7-7.6)	<0.0001	113/182	14.2 (13.0-19.3)	0.1322
nPBC	100/109	3.5 (2.8-4.6)		77/108	12.9 (9.9-16.4)	
**BRCA status**						
*BRCAm*	118/130	5.3 (4.6-6.7)	0.2937	90/130	14.3 (12.9-19.4)	0.3775
*BRCAwt*	136/157	5.7 (4.6-6.9)		96/156	13.3 (11.1-16.9)	
**Platinum-free interval**						
≤6 Months	70/73	3.0 (2.7-4.1)	<0.0001	58/73	11.2 (9.0-14.9)	0.0008
6-12 Months	78/87	6.5 (4.7-7.6)		61/87	11.9 (10.9-14.2)	
>12 Months	103/120	6.9 (5.9-8.0)		67/119	19.3 (15.3-25.7)	
**Type of initial surgery**						
Primary	84/96	6.7 (5.1-8.3)	0.0706	54/95	17.3 (14.3-25.7)	0.0006
Interval/closing	144/160	5.0 (4.2-6.0)		113/160	12.9 (11.4-15.5)	
No surgery	27/31	6.0 (4.1-9.9)		22/31	11.4 (8.7-22.7)	

1CI, confidence interval; nPBC, non-platinum-based chemotherapy; OS, overall survival; PARPi, poly(ADP-ribose) polymerase inhibitor; PBC, platinum-based chemotherapy; PFS, progression-free survival.

Among patients who received nPBC, those treated with paclitaxel had higher median PFS at 5.7 months (95% CI 4.1-7.9 months) compared with other nPBC regimens at 2.8 months (95% CI 2.6-3.5 months) (*P* = 0.0017). Outcome in the nPBC and PBC groups according to *BRCA* status and PFI are shown in Supplementary Figures S2, S3 and Supplementary Table S2, available at https://doi.org/10.1016/j.esmoop.2024.103694. We conducted then multivariable analyses to identify factors associated with better PFS with chemotherapy after PARPi exposure. PFI interval >6 months was strongly associated with PFS [adjusted HR (95% CI) 0.52 (0.39-0.70), *P* < 0.001], while interval/closing surgery compared with primary surgery was associated with worse PFS [adjusted HR (95% CI) 1.41 (1.07-1.87)]. Conversely, *BRCA* status and line of therapy for PARPi administration were not associated with PFS. Of note only 41 patients received PARPi as first-line maintenance therapy (Table 3).

**Table 3:** Univariable and multivariable Cox analyses of factors influencing PFS in whole population

	Univariable		Multivariable	
	HR (95% CI)	Cox *P*-value	HR (95% CI)	Cox *P*-value
**Whole population (*n* = 272)**				
PFI >6 months (yes versus no)	0.53 (0.40-0.70)	<0.0001	0.52 (0.39-0.70)	<0.0001
*BRCA1/2* status (mutated versus wild-type)	0.90 (0.70-1.16)	0.4245	1.05 (0.81-1.37)	0.7102
PARPi line (≥2 versus 1)	1.21 (0.83-1.77)	0.3299	1.08 (0.73-1.60)	0.6991
Type of initial surgery (ref: primary)				
Interval or closing	1.41 (1.06-1.86)	0.0169	1.41 (1.07-1.87)	0.0158
None	1.29 (0.83-2.02)	0.2598	1.29 (0.82-2.02)	0.2668

CI, confidence interval; HR, hazard ratio; PARPi, poly(ADP-ribose) polymerase inhibitor; PFI, platinum-free interval; PFS, progression-free survival.

### Outcomes of patients with subsequent chemotherapy in patients with a PFI > 6 months

As PFI was strongly associated with the PBC delivery, we conducted analyses in the group of patients with a PFI >6 months to identify the prognostic factors associated with PFS. Of 200 patients with a PFI >6 months, 159 and 41 patients received PBC and nPBC, respectively. Patients treated with PBC in this group had a numerically better PFS compared with patients receiving nPBC [adjusted HR (95% CI), 0.68 (0.46-1.01); *P* < 0.0547] (Table 4). Analyses of the group of patients were not conducted for the group of patients with PFI ≤6 months given the low number of patients receiving PBC in this group of patients.

**Table 4:** Univariable and multivariable Cox analyses of factors influencing PFS in patients with a PFI >6 months

	Univariable		Multivariable	
	HR (95% CI)	Cox *P*-value	HR (95% CI)	Cox *P*-value
**PFI >6 months (*n* = 200)**				
PBC (yes versus no)	0.70 (0.49-1.01)	0.0558	0.68 (0.46-1.01)	0.0547
*BRCA1/2* status (mutated versus wild-type)	1.01 (0.75-1.37)	0.9478	1.19 (0.86-1.66)	0.2972
PARPi line (≥2 versus 1)	1.37 (0.88-2.13)	0.1621	1.33 (0.85-2.09)	0.2115
Type of initial surgery (ref: primary)				
Interval or closing	1.32 (0.96-1.83)	0.0907	1.32 (0.95-1.83)	0.0953
None	1.24 (0.73-2.11)	0.4183	1.25 (0.73-2.14)	0.4096

CI, confidence interval; HR, hazard ratio; PARPi, poly(ADP-ribose) polymerase inhibitor; PFI, platinum-free interval.

### Patients receiving PARPi in the adjuvant setting

Among the 41 patients who received PARPi in the adjuvant setting, 35 and 6 received PBC and nPBC, respectively. The median PFS and OS were 5.5 months (4.0-10.3 months) and 20.5 months (14.9-35.8 months), as well as 6.8 months (5.1-10.3 months) and 22.7 months (19.3 months to not reached) among patients treated with PBC, respectively. Because only six patients received nPBC, no estimation was carried out in this subgroup.

## Discussion

Patients treated for ovarian cancer progression after PARPi exposure have poor prognosis, with a median PFS and OS of 5.6 and 13.6 months, respectively. Recent series indicated reduced efficacy of chemotherapy after PARPi.^15^, ^16^, ^17^, ^18^, ^19^ The survival rates of these patients were consistent with these reports. A major hypothesis for these findings is that progression under PARPi will result in subsequent chemotherapy resistance, particularly PBC.^14^ Thus, the management of this emerging population is challenging for oncologists.^2^ We found that the majority of the patients (86%) in our study received PARPi after a platinum-sensitive relapse, when maintenance was continued until progression or intolerance. The low number of patients treated with PARPi in the first-line setting reflects PARPi approvals at the time.^20^ Notably, 90% of the included patients progressed during PARPi treatment.

Our real-world study revealed that PFI was a strong prognostic factor for PFS in the whole population and patients with a PFI >6 months treated with PBC have the best outcome regardless of the number of lines received before PARPi exposure or *BRCA* status. If nPBC was proposed, weekly paclitaxel was associated with improved PFS, with a median PFS of 5.8 months (compared with 2.8 months for non-paclitaxel nPBC). Our data indicated that although patients progressing during PARPi had poor prognosis, the most active cytotoxic regimen is PBC, whereas weekly paclitaxel provides the best option among patients not eligible for PBC.

There has been a rational concern that progression following PARPi exposure may reduce subsequent chemotherapy response, particularly platinum-based regimens, as escape from PARPi, and PBC may share common resistance mechanisms.^21^^,^^22^ The restoration of HRD, by reversion mutations restoring a functional *BRCA1*/2 protein, *BRCA* hylomorphisms or loss of *BRCA1* promoter hypermethylation is the most described.^21^^,^^23^, ^24^, ^25^ Although the proportion of *BRCA1*/2 reversion mutations following platinum administration remains low (8% in the ARIEL4 trial^26^), they occur more frequently following PARPi (26.6% in a retrospective meta-analysis: 22.6% and 30.7% for *BRCA1* and *BRCA2*, respectively).^27^^,^^28^

In a retrospective series of 54 patients, PBC rechallenge after PARPi with PFI >6 months yielded a response rate of up to 25%, and four complete responses were observed in patients with PFI >12 months.^16^

The OREO trial showed that re-exposure to PARPi after PBC rechallenge in carefully selected patients resulted in a statistically significant improvement in PFS in both *BRCAm* and *BRCAwt* cohorts, although the differences were small (PFS, 4.3 versus 2.8 months in the *BRCAm* cohort and 5.3 versus 2.8 months in the non-*BRCAm* cohort).^24^

These findings indicate that mechanisms of resistance to PARPi and PBC do not necessarily overlap. We can suppose that PBC enables PARPi-specific resistance pathways related to replicative fork stabilization, specific efflux transporter activations, and other oncogenic signaling pathways to maintain sensitivity to PBC.^3^ Regarding paclitaxel results, recent studies indicate reduced *BRCA* mutation reversion after exposure, explaining its greater efficacy compared with other nPBCs.^29^^,^^30^

Notably, our study focused mainly on patients progressing under PARPi. Post-PARP outcomes were recently described in the first-line PAOLA-1 trial, which reported that patients who progressed on olaparib and bevacizumab had poorer PFS with platinum rechallenge than those progressing following PARP discontinuation.^31^

### Strengths and limitations

The international, multicenter recruitment and the broad inclusion criteria generated the largest real-life cohort of patients treated with chemotherapy after progression on PARPi. The study’s limitations include its retrospective design leading to inclusion bias, including PBC-treated patients with a longer duration of PARPi exposure and being more often *BRCA*-mutated. These unequal distributions reflect some criteria leading to PBC administration. PFI and *BRCA* mutational status are independently linked to ovarian cancer prognosis although multivariable analyses limit these biases. The timing of our cohort (before 2021) enriched our population with patients receiving PARPi in late lines, which represents a lesser population currently on PARPi. HRD status was not assessable considering the inclusion period and diversity of countries included in our study.

### Conclusion

In the absence of clear guidelines for the treatment of ovarian cancer after PARPi, practitioners choose the chemotherapy regimen. In cases of patients progressing on PARPi with a PFI >6 months, PBC rechallenge is the best option.
