# Supplementary figures and images for: Efficacy of chemotherapy after progression during or following PARPi exposure in ovarian cancer
Source: ESMO Open. 2024 Sep 3;9(9):103694. doi: 10.1016/j.esmoop.2024.103694 (PMC11403296; doi:10.1016/j.esmoop.2024.103694)

**Supplementary Material 1.** Flowchart


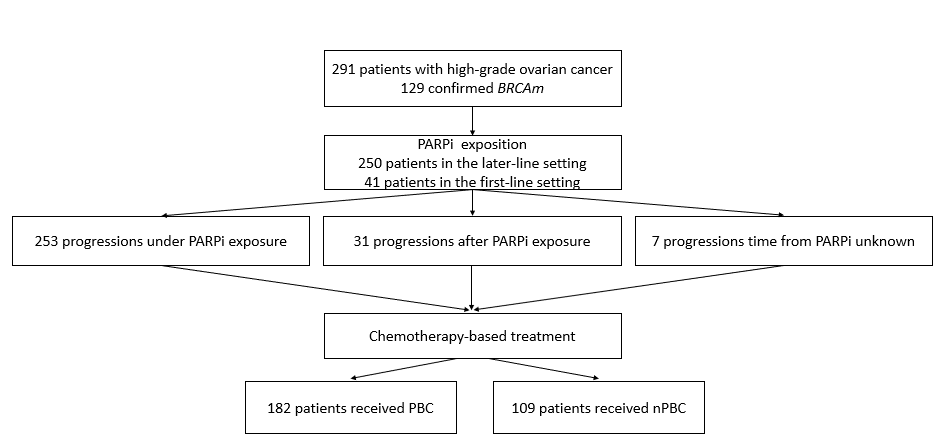

Supplement: Supplementary Material 1 [file mmc1.docx]
