# Supplementary material for: Efficacy of chemotherapy after progression during or following PARPi exposure in ovarian cancer
Source: ESMO Open. 2024 Sep 3;9(9):103694. doi: 10.1016/j.esmoop.2024.103694 (PMC11403296; doi:10.1016/j.esmoop.2024.103694)
Supplement: Supplementary Material 2 [file mmc2.docx]

**Supplementary Material 2.** Chemotherapy regimen in patients receiving nonplatinum-based chemotherapy.

|  | No. of patients |
| --- | --- |
| Cyclophosphamide | 17 |
| Doxorubicin | 24 |
| Gemcitabine | 26 |
| Ifosfamide | 1 |
| Melphalan | 1 |
| Paclitaxel | 38 |
| Topotecan | 7 |
| Trabectedin | 6 |
