# Supplementary material for: Efficacy of chemotherapy after progression during or following PARPi exposure in ovarian cancer
Source: ESMO Open. 2024 Sep 3;9(9):103694. doi: 10.1016/j.esmoop.2024.103694 (PMC11403296; doi:10.1016/j.esmoop.2024.103694)
Supplement: Supplementary Material 3 [file mmc3.pdf]

Supplementary Material 3. Kaplan–Meier curves for PFS based on the type of chemotherapy in subgroups: *BRCAm* vs. *wt* (A and B); platinum-free interval of ≤6 vs. >6 months (C and D).

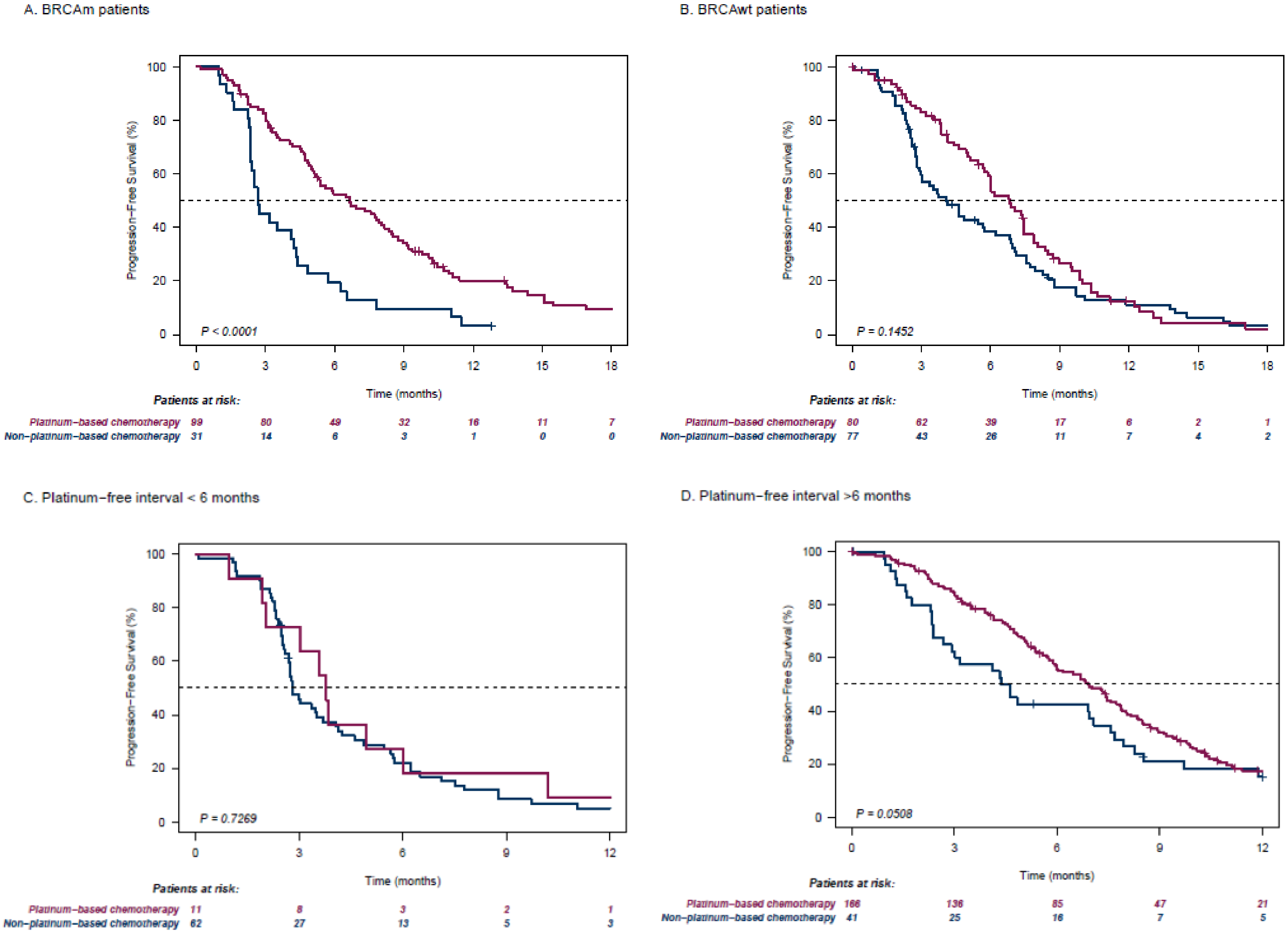
A. BRCAm patients
Progression−Free Survival (%)
100
80
60
40
20
0
P < 0.0001
0 3 6 9 12 15 18
Time (months)
Patients at risk:
Platinum−based chemotherapy 89 80 49 32 16 11 7
Non−platinum−based chemotherapy 31 14 6 3 1 0 0
B. BRCAwt patients
Progression−Free Survival (%)
100
80
60
40
20
0
P = 0.1452
0 3 6 9 12 15 18
Time (months)
Patients at risk:
Platinum−based chemotherapy 80 62 39 17 6 2 1
Non−platinum−based chemotherapy 77 43 26 11 7 4 2
C. Platinum−free interval < 6 months
Progression−Free Survival (%)
100
80
60
40
20
0
P = 0.7269
0 3 6 9 12
Time (months)
Patients at risk:
Platinum−based chemotherapy 11 8 3 2 1
Non−platinum−based chemotherapy 62 27 13 5 3
D. Platinum−free interval >6 months
Progression−Free Survival (%)
100
80
60
40
20
0
P = 0.0508
0 3 6 9 12
Time (months)
Patients at risk:
Platinum−based chemotherapy 166 136 85 47 21
Non−platinum−based chemotherapy 41 25 16 7 5
