# Supplementary material for: Efficacy of chemotherapy after progression during or following PARPi exposure in ovarian cancer
Source: ESMO Open. 2024 Sep 3;9(9):103694. doi: 10.1016/j.esmoop.2024.103694 (PMC11403296; doi:10.1016/j.esmoop.2024.103694)
Supplement: Supplementary Material 4 [file mmc4.pdf]

Supplementary Material 4. Kaplan–Meier curves for OS based on the type of chemotherapy in subgroups: *BRCAm* vs. *wt* (A and B); platinum-free interval of ≤6 vs. >6 months (C and D).

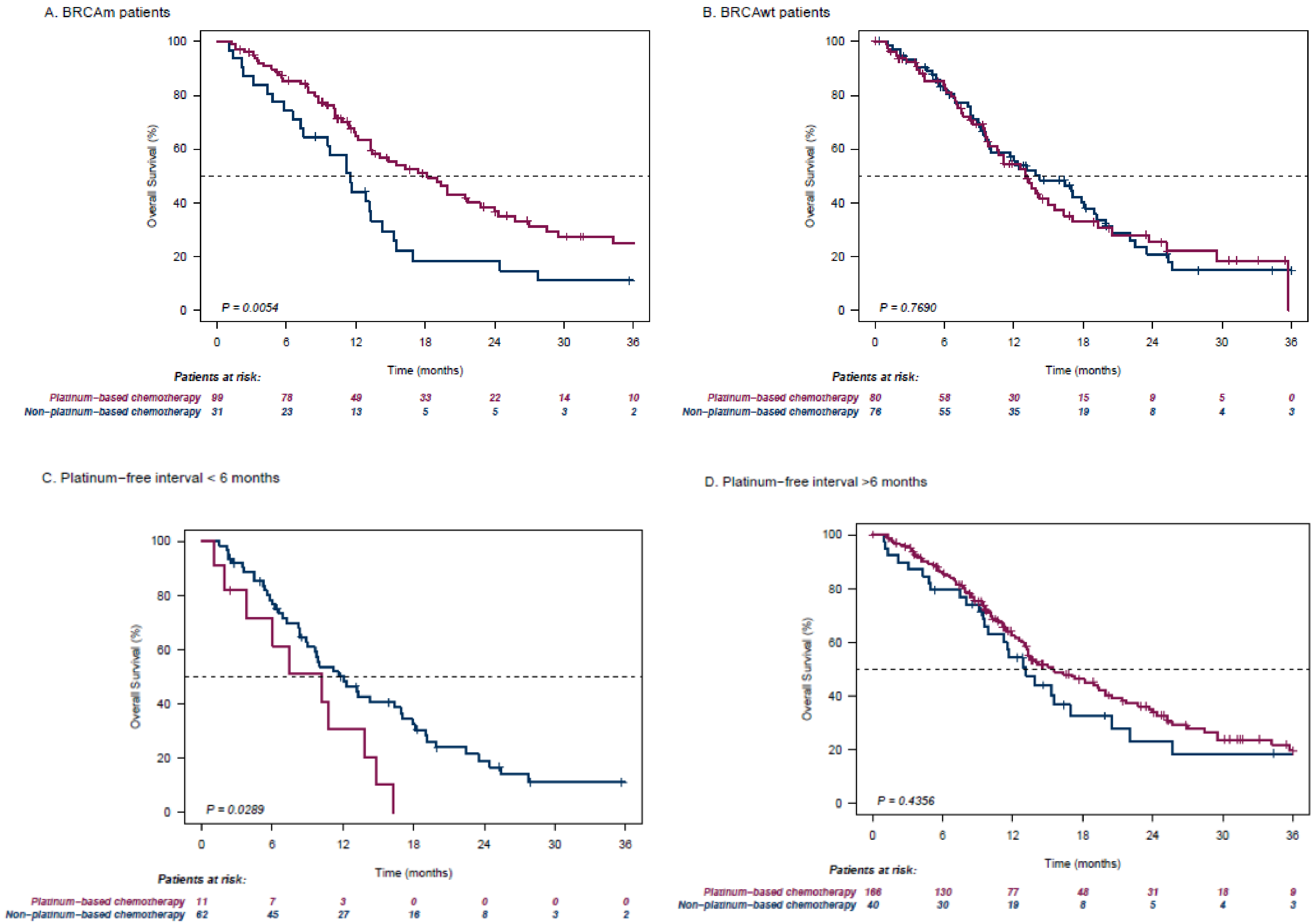

A. BRCAm patients
Overall Survival (%)
100
80
60
40
20
0
P = 0.0054
0
6
12
18
24
30
36
Time (months)
Patients at risk:
Platinum−based chemotherapy 99 78 49 33 22 14 10
Non−platinum−based chemotherapy 31 23 13 5 5 3 2
B. BRCAwt patients
Overall Survival (%)
P = 0.7690
Time (months)
Patients at risk:
Platinum−based chemotherapy 80 58 30 15 9 5 0
Non−platinum−based chemotherapy 76 55 35 19 8 4 3
C. Platinum−free interval < 6 months
Overall Survival (%)
P = 0.0289
Time (months)
Patients at risk:
Platinum−based chemotherapy 11 7 3 0 0 0 0
Non−platinum−based chemotherapy 62 45 27 16 8 3 2
D. Platinum−free interval >6 months
Overall Survival (%)
P = 0.4356
Time (months)
Patients at risk:
Platinum−based chemotherapy 166 130 77 48 31 18 9
Non−platinum−based chemotherapy 40 30 19 8 5 4 3
