# Supplementary material for: Efficacy of chemotherapy after progression during or following PARPi exposure in ovarian cancer
Source: ESMO Open. 2024 Sep 3;9(9):103694. doi: 10.1016/j.esmoop.2024.103694 (PMC11403296; doi:10.1016/j.esmoop.2024.103694)
Supplement: Supplementary Material 5 [file mmc5.docx]

**Supplementary Material 5.** Survival estimates and comparison for patients in subgroups.

|  | **PFS** | | | **OS** | | |
| --- | --- | --- | --- | --- | --- | --- |
|  | **Events/all** | **Median [95%CI] (months)** | **p (logrank)** | **Events/all** | **Median [95%CI] (months)** | **p (logrank)** |
| Type of chemotherapy following PARPi in *BRCAm* patients | | | | | | |
| *PBC* | 88/99 | 6.7 [5.2;8.3] | *<0.0001* | 62/99 | 18.2 [13.3;24.0] | *0.0054* |
| *nPBC* | 30/31 | 2.7 [2.4;4.4] |  | 28/31 | 11.5 [7.5;15.3] |  |
| Type of chemotherapy following PARPi in *BRCAwt* patients | | | | | | |
| *PBC* | 67/80 | 6.8 [5.9;7.9] | *0.1452* | 48/80 | 13.0[10.6;17.1] | *0.7690* |
| *nPBC* | 69/77 | 4.1 [3.0;6.2] |  | 48/76 | 14.3 [9.9;19.2] |  |
| Type of chemotherapy following PARPi in patients with PFI <6 months | | | | | | |
| *PBC* | 11/11 | 3.8 [3.0; NR] | *0.7269* | 10/11 | 10.2 [3.8; NR] | *0.0289* |
| *nPBC* | 59/62 | 2.8 [2.7;4.1] |  | 48/62 | 12.1 [9.0;17.1] |  |
| Type of chemotherapy following PARPi in patients with PFI >6 months | | | | | | |
| *PBC* | 144/166 | 6.9 [5.9;7.9] | *0.0508* | 101/166 | 15.5 [13.2;20.5] | *0.4356* |
| *nPBC* | 37/41 | 4.5 [2.9;7.7] |  | 27/40 | 13.1 [9.9;22.0] |  |
